# Under a Spell: Neurologic Evaluation of Presyncope as a Feature of Dysautonomia

**DOI:** 10.3390/biomedicines13112698

**Published:** 2025-11-03

**Authors:** Svetlana Blitshteyn, Kamal R. Chémali, Dennis H. Lau

**Affiliations:** 1 Department of Neurology, Jacobs School of Medicine and Biomedical Sciences, University of Buffalo, Buffalo, NY 14203, USA; 2 Dysautonomia Clinic, Williamsville, NY 14221, USA; 3 Department of Neurology, Case Western Reserve University School of Medicine, Cleveland, OH 44106, USA; kamal.chemali@uhhospitals.org; 4 Department of Cardiology, Royal Adelaide Hospital, Adelaide, SA 5000, Australia; dennis.lau@adelaide.edu.au

**Keywords:** presyncope, neurocardiogenic syncope, vasovagal syncope, postural orthostatic tachycardia syndrome, dysautonomia, spell

## Abstract

While syncope is characterized by a sudden and temporary loss of consciousness caused by decreased blood flow to the brain and is easily recognized by its clinical features, presyncope involves a sensation of impending fainting, often accompanied by autonomic symptoms. Presyncope is less characterized and studied than syncope, presenting a particular diagnostic challenge in neurology clinics. Neurologists commonly encounter patients with presyncope in outpatient settings or during consultation at the emergency department after cardiopulmonary causes have been excluded. Differential diagnosis of recurrent presyncope is broad but from a neurologic standpoint falls into multiple neurologic categories, including complex partial seizures, basilar or vestibular migraine, dysautonomia, cataplexy, alteration in cerebrospinal fluid flow, Meniere’s disease, posterior circulation transient ischemic attacks and others. Here, we review presyncope as a feature of dysautonomia and common autonomic disorders, such as neurocardiogenic syncope, postural orthostatic tachycardia syndrome, orthostatic hypotension and orthostatic intolerance. We discuss clinical and neurologic exam findings, diagnostic tests, differential diagnosis and treatment of presyncope as a manifestation of common autonomic disorders.

## 1. Introduction

While syncope is easily recognized by its defining feature of transient and self-limited loss of consciousness, presyncope as a feature of dysautonomia (which is used here to refer to autonomic disorders, such as neurocardiogenic syncope (NCS), postural orthostatic tachycardia syndrome (POTS), orthostatic hypotension (OH) and autonomic dysfunction in general) usually involves a wide range of various symptoms and signs, typically caused by or associated with decreased cerebral perfusion [1]. Presyncope can include a sensation of impending fainting, often accompanied by autonomic symptoms and may present a diagnostic challenge to neurologists because, in the absence of loss of consciousness, the symptoms reported by the patient and the signs observed by the neurologist during an episode or on a video recording may appear unusual or fall outside of classical neurologic localization. As such, misdiagnoses with psychiatric or psychological problems are not uncommon, especially when consciousness is preserved and partial seizures are ruled out [2]. In this narrative review, we discuss the limited literature on the topic of presyncope as it relates to neurologic evaluation and provide our experience as clinicians specializing in disorders of the autonomic nervous system.

Syncope is defined as a sudden and temporary loss of consciousness caused by decreased blood flow to the brain, with clinical features including rapid onset, brief duration, loss of consciousness and spontaneous recovery [3]. The lifetime cumulative incidence is high and is estimated at greater than 35% in women and men [4]. In the United States, 19% of the population will experience a syncopal event in their lifetime, with the majority occurring in young adults or after age 70 [5]. While syncope accounts for approximately 3% of visits to emergency departments [6], the incidence, prevalence and frequency of emergency visits due to presyncope are unknown and are likely much higher than those of syncope. The transient nature of symptoms without loss of consciousness and difficulty tracking such episodes in the general population contribute to a lack of data on presyncope. Furthermore, the broad scope and diverse symptomatology of presyncope present a diagnostic challenge for emergency medicine physicians, neurologists, cardiologists and other specialists who encounter patients with various types of acute or recurrent spells. Since presyncope is usually episodic and lasts minutes to hours, neurologists are often faced with a vague description of symptoms by the patient and family members who may have witnessed the episodes. Patients with recurrent presyncope are usually seen in outpatient neurology clinics when they are not experiencing presyncope, but they may also present to the emergency department during or after an acute episode, especially if it occurs for the first time, which can similarly lead to diagnostic challenges for the emergency medicine physician and consulting neurologist.

Importantly, the incidence of POTS, and dysautonomia more broadly, have been on the rise as a consequence of SARS-CoV-2 infection. One study found an increase in the incidence rate from 1.42/1,000,000 pre-pandemic to 20.3/1,000,000 cases per person-year post-pandemic, as well as an estimate of 4.21 new cases of POTS pre-pandemic to 22.66 new cases diagnosed per month after the onset of the COVID-19 pandemic [7]. Since presyncope is a common feature of POTS and post-COVID dysautonomia, clinical encounters with patients presenting with presyncope have likely increased as well, highlighting the urgent need for awareness, recognition and management of presyncope as a feature of dysautonomia.

## 2. Illustrative Case

A 24-year-old previously healthy woman developed episodes of weakness and inability to walk associated with reduced consciousness, although she never lost consciousness during the episodes. These episodes began infrequently at age 14, but increased in frequency and severity over the preceding year to now occurring several times per week. She denied loss of consciousness, postural tachycardia or chronic dizziness. In between the episodes, the patient reported mild fatigue and exercise intolerance. The episodes were precipitated by over-exertion, prolonged standing and eating dinner, after which she felt very tired and needed to lie down. She described her symptoms as “legs feeling like cement, very heavy and weak.” During the episodes, which lasted about 30 to 60 min, her blood pressure was 90/60 mmHg with a heart rate of 90 bpm; her baseline vital signs outside of the episodes were not dissimilar, with a BP of 100/64 mmHg and heart rate of 82 bpm.

Workup at the emergency department was unremarkable, and cardiac evaluation showed no evidence of cardiac arrhythmia or structural heart disease. The patient was referred for further neurologic evaluation of spells. A sleep-deprived EEG with hyperventilation was unremarkable, and MRI of the brain demonstrated no intracranial lesions or malformations. Due to complaints of muscle weakness and heavy legs, the patient had an EMG, which ruled out myopathy, neuropathy or neuromuscular junction disorders. A sleep study with mean sleep latency test was negative for narcolepsy or sleep apnea. Subsequently, a neuromuscular consult was obtained for possible hypokalemic periodic paralysis after mild hypokalemia with a serum potassium level of 3.4 was discovered on routine bloodwork. A second EMG performed by a neuromuscular neurologist was once again unremarkable. Genetic testing for hypokalemic periodic paralysis was negative. Subsequently, the patient was referred to an autonomic neurologist for further evaluation. A tilt table test showed a vasodepressor response 7 min into the tilt associated with unobtainable blood pressure, heart rate of 40 bpm with junctional rhythm, and a brief loss of consciousness. The patient was promptly returned to the supine position, at which time she regained consciousness, and her blood pressure and heart rate normalized. She was started on midodrine 5 mg three times a day along with increased fluid and salt intake, which resolved most of her episodes of presyncope within 2 weeks. However, she continued to experience mild fatigue and infrequent presyncope triggered by over-exertion and heat. This case illustrates a diagnostic challenge that led to the use of various diagnostic tests that are commonly utilized in neurology clinics for the evaluation of spells. We believe that increased awareness and recognition of autonomic disorders among neurologists may have reduced diagnostic delay and healthcare expenditure in this and many other patients presenting with presyncope. Reducing diagnostic delay and institution of the appropriate therapy for these spells with midodrine and increased fluid and salt intake could have been achieved via early recognition of presyncope as a feature of dysautonomia.

## 3. Presyncope and Autonomic Dysfunction

Although presyncope does not always mean the patient has autonomic dysfunction, recurrent presyncope in conjunction with other inter-ictal symptoms, such as chronic dizziness, fatigue, cognitive complaints (also known as “brain fog”), exercise intolerance and difficulty functioning in daily life, should prompt the neurologist to consider common autonomic disorders, such NCS, POTS, OH or autonomic dysfunction that does not fall into defined diagnostic criteria of common autonomic disorders [8,9]. While syncope defines NCS, presyncope is also common in milder cases and can be debilitating if it is frequent and recurrent, impacting the patient’s activities of daily living and quality of life. Similarly, only a subset of patients with POTS has syncope (an estimated 30%), but presyncope is a common feature of POTS along with dizziness, lightheadedness and postural tachycardia [10]. Orthostatic hypotension can manifest as frequent presyncope, syncope and orthostatic intolerance [11]. While presyncope is not a defining feature of patients with inappropriate sinus tachycardia (IST), presyncope is also a frequent complaint in that patient population, especially triggered by standing or exertion [12].

**Figure 1:**
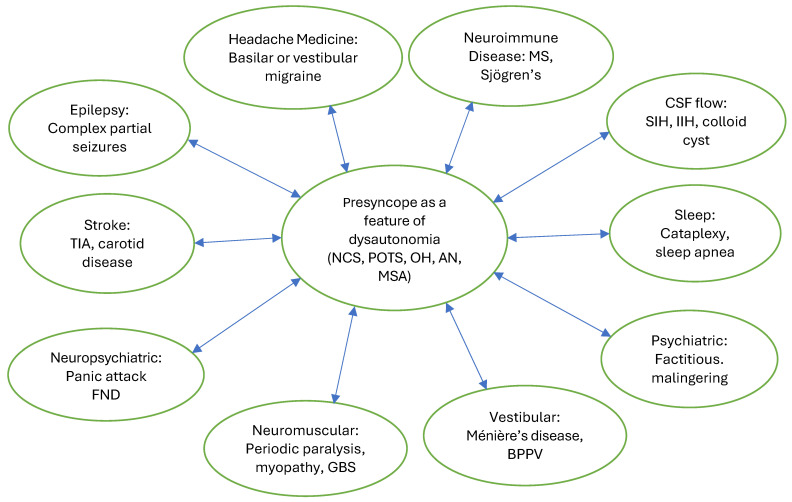
Neurologic differential c diagnosis of presyncope as a feature of dysautonomia. Abbreviations: NCS: neurocardiogenic syncope; POTS: postural orthostatic tachycardia syndrome; OH: orthostatic hypotension; AN: autonomic neuropathy; MSA: multiple system atrophy; MS: multiple sclerosis; CSF: cerebrospinal fluid; SIH: spontaneous intracranial hypotension; IIH: idiopathic intracranial hypertension; BPPV: benign positional paroxysmal vertigo; GBS: Guillain-Barre syndrome; FND: functional neurological disorder; TIA: transient ischemic attack.

Presyncope is also common in patients with small fiber neuropathy, which is one of the most common comorbidities of POTS and Long COVID [13,14]. It is also frequently reported by patients with various systemic disorders that are accompanied by comorbid dysautonomia [15]. Importantly, presyncope may be a feature of various autonomic disorders that are less prevalent in the general population than POTS, NCS and OH. These include autonomic neuropathy, including diabetic, autoimmune autonomic ganglionopathy, multiple system atrophy, pure autonomic failure and Lewy body disease. A detailed history and comprehensive neurologic examination looking for neuropathic signs, cerebellar dysfunction and Parkinsonian features, such as cogwheel rigidity, bradykinesia and decreased postural reflexes, can help narrow down the wide neurologic differential diagnosis associated with presyncope (Figure 1) and identify neuromuscular and neurodegenerative autonomic disorders among patients [16].

Pathophysiology of presyncope is not fully elucidated and likely involves multiple mechanistic factors that may closely resemble the pathophysiology of syncope, such as systemic hypotension, decreased venous return, cerebral hypoperfusion, possible altered cerebral autoregulation, baroreceptor sensitivity, hormonal alterations, immune dysregulation, vascular contractility changes and activation of vagal sensory neurons [3,17,18]. Whether presyncope and syncope exist on a spectrum of severity of cerebral hypoperfusion and other factors, or whether these factors are identical, but responses from the brain and the central autonomic networks differ, has not been determined. Future research is needed to examine the differences and similarities in mechanisms that culminate in syncope vs. presyncope.

## 4. Neurologic Evaluation

### 4.1. History

Patients describe presyncope using various subjective symptoms and descriptions (Table 1). The most common descriptors may include lightheadedness, dizziness, weakness and “feeling like I am going to faint”. Other symptoms may include general weakness, warmth, diaphoresis, nausea, palpitations, tinnitus, decreased hearing or blurry/tunnel vision. However, other neurologic complaints, such as feeling like the legs are heavy or cement-like (as described in our illustrative case), inability to move the legs, balance difficulty, unsteadiness or trouble walking, and altered consciousness or awareness are also common complaints that often prompt a neurologic evaluation.

Several key points in the patient’s history should point to presyncope occurring as a feature of dysautonomia: symptoms triggered by standing or prolonged sitting and relieved by assuming a supine position; symptoms triggered by eating a heavy meal, especially with a high carbohydrate load; symptoms triggered by heat and humidity; symptoms triggered by mild exertional activity, such as walking up one flight of stairs, walking on flat surface or dong household chores; symptoms triggered or exacerbated during or before menstruation; symptoms triggered or exacerbated by dehydration, hypoglycemia, sleep deprivation, headache or another pain; symptoms triggered or exacerbated by surgery, medical procedures physical trauma or severe and prolonged psychological stress.

Orthostatic intolerance is the hallmark of common autonomic disorders, which commonly manifests with presyncope [8,9]. Since dysfunction of the ANS can affect multiple organs and body systems, a thorough history, review of systems, and physical exam are needed to identify whether an autonomic disorder is present and which symptoms it may be causing [19]. If the patient with recurrent presyncope presents with significant cardiopulmonary symptoms that may include chest pain, shortness of breath or strictly exercise-induced or exertional presyncope, thorough cardiac and pulmonary assessment with electrocardiogram (ECG), echocardiogram, Holter or event monitoring, and appropriate tests to exclude coronary ischemia or pulmonary embolism are recommended before proceeding with neurologic evaluation.

Although a diagnostic approach to evaluation of spells has been established and has been traditionally separated into four categories, such as seizures, syncope, psychogenic, and others [20,21], neurologic evaluation of presyncope specifically has not been well-established, creating significant diagnostic uncertainty regarding which direction the neurologic evaluation should go. While research on the topic of prevalence of presyncope caused by various neurologic disorders is lacking, based on our clinical experience, we categorize neurologic evaluation of presyncope as falling in the following categories of neurologic disorders: autonomic disorders, epilepsy, headache medicine, neuromuscular disorders, stroke (especially posterior circulation TIA), sleep disorders, disorders of CSF flow, vestibular disorders, neuropsychiatric disorders and psychiatric causes (Figure 1).

### 4.2. Examination

When a patient presents with symptoms of presyncope, they may also exhibit a range of objective signs on the neurologic exam (Table 1), though in many cases, the neurologic examination may be completely normal. Objective signs can include features of autonomic dysfunction, including pallor, flushing, dilated pupils, orthostatic tachycardia hypotension or intolerance, acrocyanosis, give-way weakness, unsteady gait and others (Table 1). While give-way weakness is traditionally viewed as a sign of functional neurologic disorder (FND), we also observe this finding not uncommonly in patients with dysautonomia, which could be related to fatigue, generalized weakness and decreased effort to preserve energy during neurologic evaluation.

The neurologic examination of a patient with presyncope should begin with a 10 min stand test that measures supine and standing heart rate and blood pressure as outlined below:

1. The patient lies down quietly for 5 min; blood pressure and heart rate are obtained at the upper arm using a sphygmomanometer.
2. The patient stands up to assume an upright position without moving or talking; blood pressure and heart rate are obtained at 3, 5, 7 and 10 min of standing.
3. Patient-reported symptoms are recorded throughout the test.
4. Caution should be exercised for highly symptomatic patients who are unable to safely stand for 10 min due to orthostatic intolerance or neuromuscular disorders with impaired mobility.
5. The test can be aborted earlier than 10 min if the patient is highly symptomatic and at risk for loss of consciousness or falling.

If the 10 min stand test is consistent with a diagnosis of POTS, NCS, OH or orthostatic intolerance, then symptomatic treatment can be initiated without further confirmation via a tilt table test in a general neurology clinic or in the emergency department. However, if a 10 min stand test is inconclusive or unremarkable in a patient with suspicion for an autonomic disorder, autonomic testing that includes a tilt table test should be considered [19]. Clinicians may take into consideration any available patient-generated data from wearable heart rate devices or monitors or the patient’s self-obtained 10 min stand test performed at home [19]. These data may help with the diagnosis of an autonomic disorder when an in-office 10 min stand test is inconclusive. Note that a 10 min stand test may provide variable results depending on the time of the day, the patient’s symptoms at the time of the appointment, medication use, hydration status, medications and other factors. When the diagnosis is uncertain or symptoms are progressing, a referral to an autonomic specialist should be considered [19].

At least 50% of patients with POTS have small fiber neuropathy, which warrants a careful neurologic sensory examination, including pinprick and temperature sensation, to help identify small fiber neuropathy [22]. Other potential signs of autonomic dysfunction include abnormal pupillary exam with dilated pupils that are poorly responsive to light, and evidence of acrocyanosis—a purplish-blue discoloration of the upper and lower extremities due, in part, to blood pooling [23]. Acrocyanosis may also occur in patients with Raynaud’s disease, other connective tissue disorders and erythromelalgia and may sometimes point toward an autoimmune etiology [24]. Assessment for joint hypermobility with the Beighton scale is recommended because hypermobility spectrum disorders and hypermobile Ehlers–Danlos syndrome (EDS) are highly prevalent in patients with presyncope, vasovagal syncope and POTS [25]. Similarly, flushing, urticaria and dermographism may be present on skin examination of patients with presyncope because mast cell hyperactivity is a common comorbid condition in patients with dysautonomia, and in fact, may be one of the main drivers of presyncope [26]. A detailed cardiovascular exam is also warranted (Table 2).

### 4.3. Diagnostic Tests

Since presyncope has a broad differential neurologic diagnosis (Table 3) that may fall into 10 categories of neurologic subspecialties (Figure 1), the history and neurologic exam may aid in selecting the appropriate diagnostic tests to arrive at the correct diagnosis. Diagnostic tests that are commonly utilized as part of the neurologic assessment may include an MRI of the brain to rule out structural causes, including a colloid cyst of the third ventricle and other lesions that may obstruct CSF flow and cause syncope and presyncope [27], a structural lesion that could be epileptogenic or an neuroimmune disease, such as multiple sclerosis, that could lead to autonomic dysfunction. A sleep-deprived EEG with hyperventilation to rule out complex partial seizure disorders that may cause spells with preserved consciousness [28], and a sleep study to rule out narcolepsy that can cause daytime cataplexy with preserved consciousness may be necessary [29]. Additionally, undiagnosed and untreated central or obstructive sleep apnea may worsen presyncope that is caused by autonomic dysfunction. Other possible diagnostic tests that may be beneficial in patients with recurrent presyncope are autonomic function tests to assess for autonomic neuropathies, small fiber neuropathy and central autonomic disorders; EMG to rule out large fiber neuropathy and other neuromuscular disorders that may manifest as presyncope and episodic muscle weakness; a skin biopsy to assess for small fiber neuropathy—a common comorbidity in patients with autonomic disorder; an exercise stress test to rule out coronary artery disease and chronotropic incompetence; rhythm monitoring to assess for recurrent cardiac arrhythmia, cardiac MRI to evaluate for possible structural abnormalities, and supine and standing transcranial Dopplers to determine whether orthostatic cerebral hypoperfusion is present if available. Supine and standing serum catecholamine levels can also be helpful in identifying a hyperadrenergic state that can accompany certain phenotypes of POTS. CBC, CMP, TFT, ferritin, morning cortisol, vitamin B12, methylmalonic acid and homocysteine, CRP, ESR, ANA and other more specific antibodies, such as a celiac panel, Sjögren’s antibodies, thyroid antibodies, anti-parietal cell antibody as well as ganglionic nicotinic acetylcholine receptor antibodies and voltage-gated calcium and potassium channel antibodies can also be obtained in certain patients if warranted [9,10,11,19]. If access to an autonomic laboratory is available, patients with questionable diagnosis, progressing disease course and treatment-refractory symptoms should be referred for studying the branches of the autonomic nervous system that are not detected by a 10 min stand test or a tilt table test. Unfortunately, given the paucity of these specialized laboratories in the United States and other countries, at least a 10 min stand test or a tilt table test are necessary for evaluation of presyncope.

### 4.4. Etiology

The etiologies of presyncope are diverse, ranging from outpatient conditions to medical emergencies [1]. Presyncope can be caused by the same underlying etiologies as syncope and include neurologic, cardiac, pulmonary, metabolic, endocrine and other etiologies [30]. Cardiac presyncope, which is caused by cardiac arrhythmia, is commonly associated with palpitations, shortness of breath and irregular heart rhythm and is usually non-positional. Presyncope triggered by exercise and exertion in general warrants a prompt cardiovascular evaluation to rule out cardiac causes. When presyncope is new or occurs in conjunction with other symptoms, such as chest pain, shortness of breath or focal neurologic signs, an emergency workup is necessary to rule out myocardial infarction, pulmonary embolism, aortic dissection, vertebral dissection, transient ischemic attacks and other medical emergencies that can present with syncope or presyncope [1,3,30].

After cardiopulmonary and neurologic emergencies, such as myocardial infarction, pulmonary embolism, aortic dissection, vertebral dissection, and transient ischemic attacks are ruled out, outpatient medical and neurologic assessments for presyncope are necessary. Medical assessment usually includes ruling out dehydration, hypoglycemia, iron-deficiency anemia, electrolyte abnormalities, thyroid disorders and medication side effects, which can be caused by antihypertensives, cardiovascular agents, antidepressants and antipsychotics. Substance use should also be assessed, especially alcohol, which is a vasodilator. Neurologic assessment is typically obtained for complaints of generalized weakness, pain, discomfort or a heavy sensation in the legs, unsteady gait, dizziness, altered consciousness or awareness and recurrent shaking or tremulousness. These complaints often prompt a neuromuscular evaluation to rule out paroxysmal neuromuscular disorders; epilepsy consults and monitoring to rule out seizures; headache evaluations for possible basilar and vestibular migraine; sleep evaluations to rule out cataplexy and neuropsychiatric assessment to determine if functional neurologic disorder, factitious disorder or a mood disorder is present (Figure 1).

### 4.5. Treatment

Although treatment of presyncope depends on etiology, when it occurs as a part of autonomic dysfunction, treating dysautonomia is imperative. First, discontinuing or reducing medications that can cause presyncope is warranted, and substances, such as alcohol, need to be eliminated. Second, improving systemic and cerebral perfusion by achieving blood volume expansion and minimizing blood pooling into the lower abdomen or the lower extremities is needed. This is achieved by an increased fluid intake of 2.5 to 4 L per day and salt (sodium chloride intake) of at least 7 g per day, unless the patient has hypertension, kidney or cardiac disease that precludes a high sodium consumption. Wearing compression garments, including waist-high compression stockings and abdominal binders, might be helpful in reducing blood pooling in the lower extremities and lower abdomen, respectively [31]. Elevating the head of the bed to 70 degrees may decrease nocturia and help with morning dizziness and presyncope by reducing the release of atrial natriuretic peptide, thereby reducing hypovolemia. Voluntary leg contractions and counter-maneuvers to increase muscle pump and improve venous return can be helpful in some patients [32], but can worsen symptoms in others. We advise patients with presyncope and a high risk of syncope and falls to avoid syncope and sit down or lie down when presyncope ensues. Avoiding heat and humidity as much as possible is also recommended because many patients have heat intolerance and will experience presyncope when the ambient temperature is high. Exercising in a seated position or in the pool with water jogging is preferred, although some people with presyncope will be able to tolerate more intense aerobic exercise [33]. If presyncope is exercise-induced, a clearance from a cardiologist is necessary prior to resuming exercise. Finally, aside from a high-sodium diet, gluten-free low-carb diet may be beneficial in some patients with POTS [34]. Gluten can be pro-inflammatory, and many patients with dysautonomia have gluten sensitivity, while a high carbohydrate load can trigger blood pooling and release of glucose-dependent insulinotropic polypeptide that can promote abnormal glucose metabolism and vasodilation [35].

Pharmacotherapy for symptomatic management of presyncope can include midodrine, an alpha 1 agonist and vasoconstrictor, pyridostigmine, an acetylcholinesterase inhibitor that enhances parasympathetic transmission; propranolol or atenolol, beta blockers that reduce tachycardia and sympathetic overactivity; ivabradine that decreases tachycardia through I-channels; fludrocortisone, an aldosterone analog that enhances sodium resorption; droxidopa, which is FDA-approved for neurogenic orthostatic hypotension; desmopressin that improves hypovolemia; stimulants such as methylphenidate, amphetamine/dextroamphetamine salts or lisdexamfetamine dimesylate, which act as vasoconstrictors; and venlafaxine or atomoxetine, which increase norepinephrine in the brain [19,32]. Clonidine and methyldopa, which are central sympatholytics, may be utilized in some patients with presyncope associated with hyperadrenergic state, tachycardia and blood pressure elevation, but these can also cause hypotension and syncope as adverse effects, and therefore, could be considered on a case-by-case basis. Additionally, intravenous saline may be beneficial, especially during acute exacerbation of presyncope or in patients who have difficulty increasing fluids and salt intake due to nausea, gastroparesis or mast cell activation syndrome (MCAS) [36]. Finally, comorbidities of presyncope as part of dysautonomia should be identified and treated appropriately. These comorbidities include autoimmune disorders, small fiber neuropathy, metabolic disorders, migraine, neuropathic pain, sleep disturbance, chronic joint pain from possible hypermobility spectrum disorders and reactions to food, medications and environmental triggers as part of MCAS [25,37]. Similarly, if psychiatric comorbidities, such as generalized anxiety disorder, panic disorder, depression, OCD, ADHD or FND are present, they should be treated accordingly with psychological therapies and medication if necessary. Hormonal abnormalities if present, such as hypothyroidism, premenstrual syndrome, perimenopause or low testosterone levels, should also be addressed as these may cause or contribute to autonomic dysfunction, including presyncope. If presyncope, orthostatic intolerance and dysautonomia are severe and treatment-refractory, especially if associated with elevated markers of inflammation or autoimmunity, immunotherapies can be considered [38].

## 5. Considerations in Psychiatry, Psychology and Physician–Patient Communication

Many patients with presyncope may describe episodic symptomatology highly suggestive of a panic attack. It is important for neurologists not to jump to conclusions that symptoms are due to a panic attack or are psychogenic in nature and consider dysautonomia as the cause of presyncope first [2]. While in a subset of patients, anxiety and other neuropsychiatric symptoms can accompany autonomic and other neurologic disorders, such as migraine, epilepsy and multiple sclerosis, misattribution of presyncope to generalized anxiety, depression, panic disorder or FND are common in clinical practice, which leads to a missed diagnosis and opportunity for identification and treatment of the underlying autonomic disorder [2]. For example, in one study of 3471 patients with POTS, 75% of patients reported having been misdiagnosed by a physician prior to a diagnosis of POTS, with 77% encountering a misdiagnosis with a psychiatric or psychological problem before their POTS diagnosis [37]. The high prevalence of misdiagnosis among patients with dysautonomia results in inappropriate or delayed care, wasted resources, persistent disability from an undiagnosed and untreated disorder and a detrimental physician-patient relationship, which can lead to patients developing secondary psychological problems and medical PTSD. Finally, poor quality of life and significant socio-economic burden faced by patients with POTS, with more than 50% being unable to maintain employment and 70% losing income due to POTS, may lead to psychological, physical and financial stress as well as economic and societal adversities [39].

Effective communication and education of patients is an essential part of the neurologic evaluation to encourage trust in the healthcare system and improve compliance, treatment outcomes and quality of life in patients with autonomic disorders and other complex chronic illnesses [40]. Communication strategies should avoid phrases that blame the patient’s mental, hormonal or fitness state for their disabling chronic symptoms and functional impairment [40]. While there are no studies on the impact of frequent and recurrent presyncope, studies on NCS show a 30% recurrence rate that can lead to significant morbidity and reduced quality of life [41]. More than half of patients with POTS are unemployed with disability comparable to patients with CHF and COPD [39,42]. Although traditionally NCS and presyncope have been viewed as “benign,” we recommend against using this description when communicating the nature of their problem, especially in patients with frequent recurrent presyncope causing significant functional impairment [40].

A common misconception that exists among neurologists is that dysautonomia may be associated with or based in functional neurological disorders (FND). It is important to emphasize that autonomic disorders, including POTS and NCS, are not FND and are not caused by functional etiology [2,43]. Although a few patients may have the so called “psychogenic pseudosyncope/presyncope”—a form of FND—either as the sole explanation of presyncope or in conjunction with NCS or POTS [44], the pseudosyncope is likely a byproduct of superimposed psychological distress, possible poor coping mechanisms, inadequate healthcare and other biopsychosocial factors that can be associated with a chronic and debilitating medical condition. In these rare cases where both dysautonomia-related presyncope and psychogenic pseudosyncope co-exist, treatment of dysautonomia should be initiated first before implementation of FND-tailored physical therapy [2].

## 6. Future Directions

Presyncope is commonly encountered in clinical practice, but research studies on recognition, differential diagnosis and treatment of presyncope are lacking. Most studies and consensus guidance statements focus on syncope, POTS and orthostatic hypotension, with no mention of presyncope as a diagnostic entity. Further research and addition of presyncope to the broad scope of autonomic problems, as well as identification of diagnostic biomarkers for presyncope and syncope, are needed to improve diagnosis, classification and treatment options in patients experiencing presyncope as a feature of dysautonomia.

## 7. Key Points

- Unlike syncope where loss of consciousness leads to an established diagnostic and therapeutic approach, presyncope is harder to recognize and is less studied. Because there is no loss of consciousness, its prevalence and incidence in the general population or patients presenting to neurology clinics are unknown; the scope of presyncope symptoms and signs is broad, presenting a diagnostic challenge.
- New-onset presyncope may warrant an urgent diagnostic workup in the emergency department to rule out medical emergencies, including cardiac, pulmonary, neurologic and metabolic disorders.
- After medical emergencies have been ruled out, recurrent presyncope has a broad differential neurologic diagnosis: neurologists should consider presyncope as a feature of dysautonomia after cardiac arrhythmia, other structural cardiac abnormalities and complex partial seizures have been ruled out.
- Detailed history may include orthostatic dizziness, fatigue and exercise intolerance in addition to presyncope triggered by standing, prolonged sitting, mild exertion, heavy meal, heat, dehydration or menstrual symptoms.
- Neurologic exam with a 10 min stand test should be performed at the initial neurologic evaluation to avoid misdiagnosis and diagnostic delay and optimize appropriate therapies for autonomic dysfunction and treatment outcomes.

## Figures and Tables

**Table 1:** Clinical features of presyncope.

Subjective Symptoms and Description	Possible Objective Findings and Observations
Lightheadedness	Pallor or flushing
Dizziness	Slow gait
Near-fainting	Unsteady gait
About to fall over	Tremulousness
Imbalance	Perspiration
Unsteadiness	Rapid breathing
Weakness	Distressed appearance
Palpitations	Anxious appearance
Racing heart	Acrocyanosis
Shortness of breath	Blue lips
Headache	Tachycardia
Tunnel vision	Bradycardia
Blackout vision	Faint pulse
Difficulty concentrating	Hypotension
Dreamlike state	Hypertension
Derealization and depersonalization	Narrow pulse pressure
Spaced out feeling	Give-way weakness
Disconnected feeling	Fine postural tremor
Nausea	Hyperreflexia
Dyspnea or hyperventilation	Hypotonia
Numbness or tingling	Hypertonia
Heavy or cement legs	Shivering
Feeling like the legs cannot move	Piloerection
Sleepiness	Dilated pupils

**Table 2:** Neurologic evaluation of recurrent presyncope.

Orthostatic vital signs
A 10 min stand test
Pulse oximetry
Neurologic exam
**Neurologic tests**: MRI of the brain, sleep-deprived EEG with hyperventilation, tilt table test and other autonomic function tests if available, polysomnography with mean sleep latency test
**Cardiac tests**: ECG, cardiac echo, 48-Holter monitor, 30-day cardiac event monitor
**Possible other diagnostic tests**: EMG, skin biopsy for small fiber neuropathy, MRA of the head and neck, MRV, exercise stress test, implantable loop recorder, cardiac MRI, supine and standing transcranial Dopplers, minor salivary gland biopsy to rule out Sjögren’s disease, audiogram to rule out Ménière’s disease
**Labs**: CBC, CMP, TFT, ferritin, morning cortisol, vitamin B12, methymalonic acid, homocysteine, vitamin B6, immunofixation serum and urine, CRP, ESR, RF, ANA
**Possible other labs**: Sjögren’s antibodies, thyroid antibodies, celiac panel, anti-parietal antibodies, ganglionic AchR antibodies, voltage-gated calcium and potassium channel antibodies, supine and standing serum catecholamines, plasma metanephrines and urine fractionated metanephrines, 24 h urine 5-HIAA and porphobilinogens, serum tryptase and histamine, LH, FSH, testosterone, estrogen, insulin, C-peptide, serum and urine mitochondrial biomarkers

**Table 3:** Differential diagnosis of recurrent presyncope.

Neurologic:
Seizure
Transient ischemic attack
Migraine with aura
Basilar migraine
Vestibular migraine
Transient migraine accompaniments
Cataplexy
Spontaneous intracranial hypotension
Idiopathic intracranial hypertension
Cranio-cervical instability
Hypokalemic periodic paralysis
Vestibular dysfunction
Meniere’s disease
Pain-induced
Cardiopulmonary:
Cardiac arrhythmia
Cardiac conduction disorder
Coronary arterial disease
Cardiomyopathy
Cardiac valvular disease
Pulmonary embolism
Pulmonary hypertension
Hyperventilation
Endocrine:
Hypoglycemia
Thyrotoxicosis
Pheochromocytoma
Adrenal insufficiency
Menopausal hot flashes
Pregnancy
Testosterone deficiency
General medical:
Medication side effect
Anemia
Dehydration
Overheating
Infection
Substance use
Allergic reaction
Labile hypertension
Orthostatic hypotension
Porphyria
Psychiatric:
Panic attack
Anxiety
PTSD
Functional Neurologic Disorder
Factitious disorder
Malingering

## Data Availability

No new data were created or analyzed in this study. Data sharing is not applicable to this article.
